# MicroRNA Expression Profile Analysis in Blood During Giant Panda (*Ailuropoda melanoleuca*) Growth and Development

**DOI:** 10.3390/genes16030243

**Published:** 2025-02-20

**Authors:** Shanshan Ling, Die Deng, Fuxing Yang, Pingfeng Wang, Ming He, Qian Wang, Linhua Deng, Xun Wang, Ling Zhao, Gang Ye, Xiaoyu Huang

**Affiliations:** 1 China Conservation and Research Centre for the Giant Panda, Dujiangyan, Chengdu 611800, China; lingshanshan86@163.com (S.L.); wangpingfeng2017@163.com (P.W.); hemingxx619@163.com (M.H.); wqian1295@163.com (Q.W.); pandayak@163.com (L.D.); 2 College of Animal Science and Technology, Sichuan Agricultural University, Chengdu 611130, China; 18180635647@163.com (D.D.); 18884328121@163.com (F.Y.); wangxun99@163.com (X.W.); 3 College of Veterinary Medicine, Sichuan Agricultural University, Chengdu 611130, China; lingzhao@sicau.edu.cn (L.Z.); yegang800206@163.com (G.Y.)

**Keywords:** giant panda, miRNA, blood, development, immunity

## Abstract

Background/Objectives: Blood is an essential component of the immune system. As post-transcriptional regulators, miRNAs, abundant in blood, are necessary aspects in blood’s immune and physiological functions. However, there is limited knowledge about the expression and function of miRNAs in the blood of giant pandas. Methods: We comparatively analyzed miRNA expression profiles in the blood of giant pandas of different ages using small-RNA sequencing technology. Results: We identified 393 known miRNAs, 219 conserved miRNAs, and 71 novel miRNAs in the blood of giant pandas, and functional enrichment analysis showed that the genes regulated by DE (differentially expressed) miRNAs were mainly enriched in the regulation of enzyme-linked receptor protein signaling pathways and the signaling pathways of MAPK, Hippo, and FoXO. Conclusions: Our study clarified giant pandas’ blood miRNA expression profiles at different developmental stages, which will help elucidate the blood immunity and regulation of blood cell physiological functions in giant pandas.

## 1. Introduction

The giant panda holds significant cultural and ecological value as a national treasure of China and a rare animal endemic to the country, attracting widespread attention [1]. In the past, the giant panda faced severe threats due to the loss of its habitat caused by deforestation, resulting in its endangered status [2]. Fortunately, in recent years, with the dedicated efforts of Chinese scientists and conservation managers, the giant panda, as a flagship species with significant ecological and scientific value, has made remarkable progress in population recovery and growth [3].

Blood is a “liquid connective tissue” in which blood cells constantly interact with tissue cells throughout the body [4]. It is an easily accessible sample that can be used as an alternative to tissue samples for various molecular analyses. Blood plays a crucial role in immunity, inflammation, and maintaining physiological homeostasis, and blood cell profiling is a powerful tool for exploring disease pathogenesis and physiological homeostasis [5]. Genome-wide assessment of changes in transcript abundance in blood provides a powerful tool for studying variations in the immune system during health and disease [6]. Furthermore, due to the rapid turnover rate of blood cells, subtle cellular changes in tissues associated with injury or illness can trigger specific changes in gene expression at the microscopic level within blood cells. These changes can be used as biosensors for diagnostic purposes [7]. Considering the importance of changes in immune function during growth, development, and aging, it is essential to examine changes in the blood of giant pandas at different ages [8].

MicroRNAs (miRNAs) are non-coding RNAs of approximately 21 nucleotides in length that serve as key post-transcriptional regulators of gene expression [9]. In the RNA-induced silencing complex, one strand of a mature miRNA binds to the target mRNA’s 3′-untranslated region (3′-UTR) through incomplete base complementarity, leading to transcriptional repression or induced mRNA degradation [10]. In a wide range of biological processes, such as development, cell differentiation, proliferation, and apoptosis, miRNAs play crucial roles as regulators of gene expression [11].

MiRNAs are small, molecularly stable, and abundant in blood. They can be measured both in blood cells and serum circulation. So, miRNA control is a key regulatory mechanism of the immune system and represents a potential source of biomarkers. Serum or plasma miR-21 is overexpressed in tumors of the stomach, ovary, brain, biliary tract, and liver, and thus, miR-21 may serve as a useful blood-based biomarker for the diagnosis of various types of human cancers [12]. miR-17-92 and -106a-363 clusters were linked with the obesity-associated low type-2 asthma phenotype [13]. MiR-155 expression has been associated with several leukemia-associated phenotypes and poor prognosis of hematologic malignancies, and its overexpression may serve as a reliable indicator of unfavorable prognosis [14]. MiR-122 blood levels correlate with the severity of cardiovascular disease [15]. Plasma levels of miRNAs have also been associated with telomere length, fertility, and blood metabolites related to body energy balance and metabolic stress [16]. miR-1908-5p has been found to play an essential role in the regulation of low-density lipoprotein (LDL), total cholesterol (TC), fasting glucose (FG), glycated hemoglobin (HbA1c), and several lipid metabolites in blood [17]. Zhang et al. found that the expression levels of six miRNAs, namely miR-362, miR-221-3p, miR-210, miR-30a-5p, miR-30a-3p, and miR-26a, in porcine peripheral blood were negatively correlated with age and were overexpressed in immune-related pathways (e.g., leukocyte migration, viral processes) [18].

To date, miRNAs have been identified in the blood of various animals, such as rhesus monkeys [19], pigs [20], and cows [21]. Wang et al. discovered conserved miRNAs in the peripheral blood of three giant pandas during late gestation and early lactation [22]. Similarly, Yang et al. identified and compared miRNAs in the blood of two subadult and two adult giant pandas [23]. However, there has been no previous investigation of miRNAs in the blood of pandas younger than two years of age. In this study, we examined the miRNA expression profiles of nine giant pandas across three age stages: subadult pandas aged 1.5 to 2 years, subadult pandas aged 2.5 to 3 years, and adult pandas over eight years old. We aimed to identify miRNAs and explore their potential role in giant pandas. The results will be helpful in understanding the blood immune function and blood cell physiology of giant pandas from the perspective of miRNAs in a wider age range and will provide abundant experimental data.

## 2. Materials and Methods

### 2.1. Animals and Samples

This study utilized blood samples from giant pandas collected from the China Conservation and Research Center for the Giant Panda. Nine giant pandas were included: three subadult pandas aged 1.5 to 2 years (Subadult(1.5Y–2Y)), three subadult pandas aged 2.5 to 3 years (Subadult(2.5y–3Y)), and three adult pandas aged eight, twelve, and fifteen years old (Adult).

### 2.2. RNA Extraction and High-Throughput Sequencing

Blood samples from nine giant pandas across different age stages were used for total RNA extraction with RNAiso Blood reagent. The quantity of total RNA samples was assessed using an Agilent 2100 Bioanalyzer (Agilent Technologies, Santa Clara, CA, USA). Each giant panda blood RNA sample was used to construct a small-RNA library. For each library, small RNAs ranging from 15–36 nt in length were separated from total RNA by polyacrylamide gel electrophoresis and ligated with proprietary adaptors. Single-stranded cDNA was synthesized according to Illumina’s recommended reverse transcription primers and amplified by PCR. High-throughput sequencing was performed by Novogene using the Nova-SE50 platform.

### 2.3. Identification and Differential Expression Analysis of miRNAs

Before identifying the miRNAs of giant pandas, the initial sequences needed to be strictly filtered. The outputs are called clean reads after removing low-quality reads, repetitive sequences, and adaptors. The filtered sequences were aligned with the giant panda genome (*Ailuropoda melanoleuca*. ASM200744v2) using Bowtie2 v2.4.5 software under criteria that allowed for one mismatch. Such sequences in the comparison are called mappable reads. Since there are no annotations related to giant panda miRNAs in miRBase 22.0, known miRNAs are obtained by aligning the mappable reads’ extended gene sequences with mammalian miRNA sequences in miRBase 22.0. Conserved miRNAs are identified by aligning them with miRNA sequences of vertebrates other than mammals, and novel miRNAs by using the miRDeep2 core algorithm to predict the new miRNAs.

Differential miRNA expression analysis across ages was performed with the OmicShare tool using the EdgeR v.4.4.2 software package (http://www.omicshare.com/tools, accessed on 18 August 2023). In this study, the screening criterion for differential expression was that miRNAs were considered differentially expressed only when the value of |log2 Fold Change| > 1 and the FDR (False Discovery Rate) < 0.05.

### 2.4. Target Gene Prediction and Functional Enrichment Analysis

Target gene prediction of the screened DE miRNAs was performed using the TargetScan algorithm (https://www.targetscan.org/, accessed on 1 September 2023), and the resulting target gene set was then entered into the Metascape website (http://www.metascape.org/, accessed on 1 September 2023) for the analysis of gene ontology (GO) and the KEGG pathway enrichment analysis.

## 3. Results

### 3.1. Overview of miRNA Sequencing Data

To identify miRNAs and study the expression changes of miRNAs during the development of giant pandas, total RNAs were extracted from the blood of giant pandas at three different ages. These RNAs were then used to construct nine small-RNA libraries. High-throughput sequencing was used to sequence all of the constructed libraries, generating a total of 118.28 million raw reads. Adaptor sequences, contaminants, and low-quality reads were removed; the remaining reads were considered high-quality clean reads. The proportion of high-quality clean reads for the nine libraries ranged from 90.4% to 95.4% (Table 1). Furthermore, an analysis of length distribution revealed that most small RNAs (63.6–93.9%) exhibited length peaks between 20 and 24 nt (Figure S1). This length distribution pattern aligns with the typical characteristics of animal miRNAs.

### 3.2. Identification of miRNAs and Expression Profiles in Blood of Giant Pandas of Different Ages

We identified 683 mature miRNAs from 1044 pre-miRNAs in the blood of giant pandas of three ages (Table 2). These candidate miRNAs were categorized into three types: known miRNAs (Table S1), conserved miRNAs (Table S2), and putative novel miRNAs (Table S3). A total of 393 known miRNAs in giant pandas corresponded to 314 pre-miRNAs. In total, 219 conserved miRNAs in giant pandas corresponded to 643 pre-miRNAs from other mammalian species. Finally, 71 putative novel miRNAs corresponded to 87 candidate pre-miRNAs.

**Figure 1:**
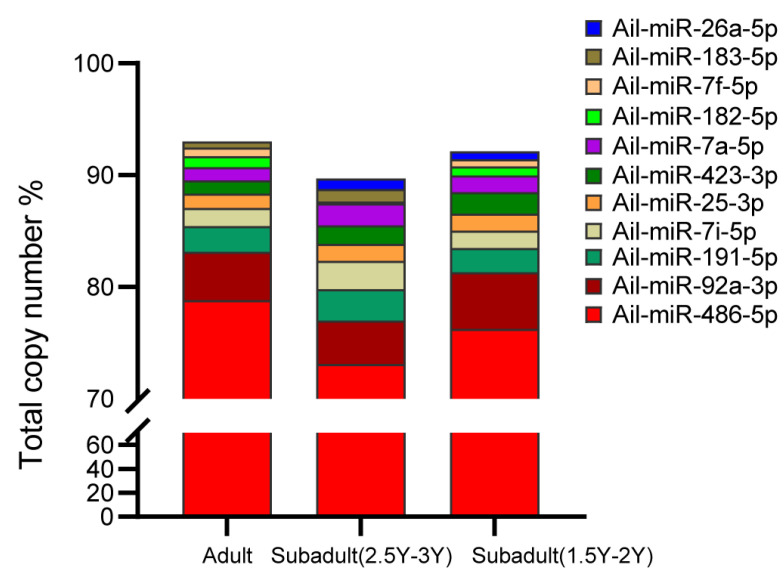
The top 10 unique miRNAs with the highest expression in the blood of giant pandas at different age stages.

To explore the potential roles of miRNAs in the blood of giant pandas at different ages, identified miRNAs were ranked based on their expression abundance. Figure 1 illustrates that the top 10 unique miRNAs accounted for 91.2%~93.0% of the total counts for each development stage. The unified set of the top 10 unique miRNAs in the three stages corresponded to 11 unique miRNAs. Among these miRNAs, nine (Ail-miR-486-5p, Ail-miR-92a-3p, Ail-miR-191-5p, Ail-miR-7i-5p, Ail-miR-25-3p, Ail-miR-423-3p, Ail-miR-7a-5p, Ail-miR-7f-5p, Ail-miR-183-5p) were found to be consistently expressed across all stages.

### 3.3. Differential Expression (DE) miRNA Analysis

**Figure 2:**
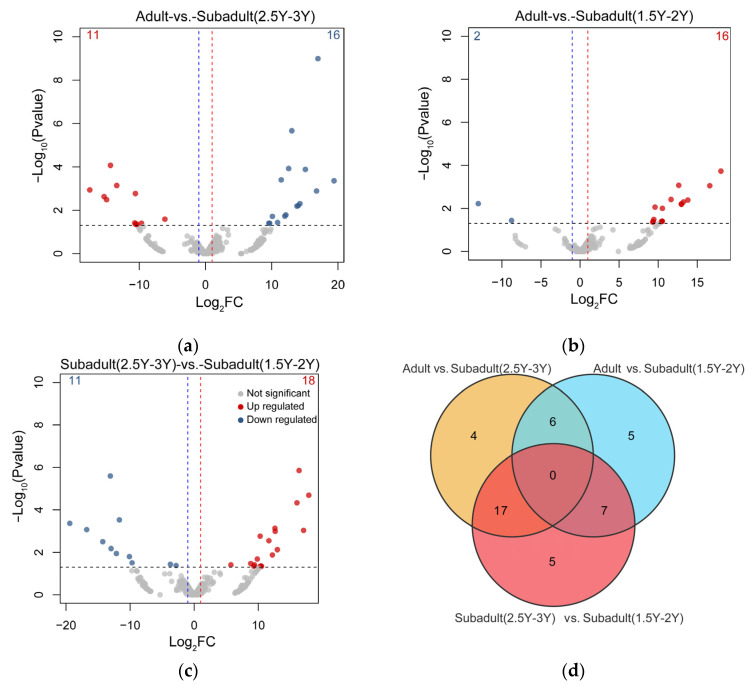
Differential miRNA analysis between two different age groups: (**a**) Adult vs. Subadult (2.5Y–3Y); (**b**) Adult vs. Subadult (1.5Y–2Y); (**c**) Subadult (2.5Y–3Y) vs. Subadult (1.5Y–2Y); (**d**) Venn plot revealing that differentially expressed miRNAs exist in the three different age stages. Note: 1.5Y–2Y: 1.5–2 years old; 2.5 Y–3 Y: 2.5–3 years old.

To identify differentially expressed (DE) miRNAs at different ages, we conducted differential expression analysis using the criteria of |log2(fold change)| > 1 and FDR < 0.05 after removing miRNAs with fewer than 10 counted reads. We identified 44 DE miRNAs, accounting for 6.44% of the total identified miRNAs (Table S4). Notably, a comparison of Adult vs. Subadult(2.5Y–3Y) (Figure 2a), Adult vs. Subadult(1.5Y–2Y) (Figure 2b), and Subadult(2.5Y–3Y) vs. Subadult(1.5Y–2Y) (Figure 2c) identified 27, 18, and 29 DE miRNAs, respectively (Figure 2). However, the Venn diagram of DE miRNAs (Figure 2d) indicates that no miRNA was differentially expressed across all three developmental stages.

### 3.4. Functional Enrichment Analysis

**Figure 3:**
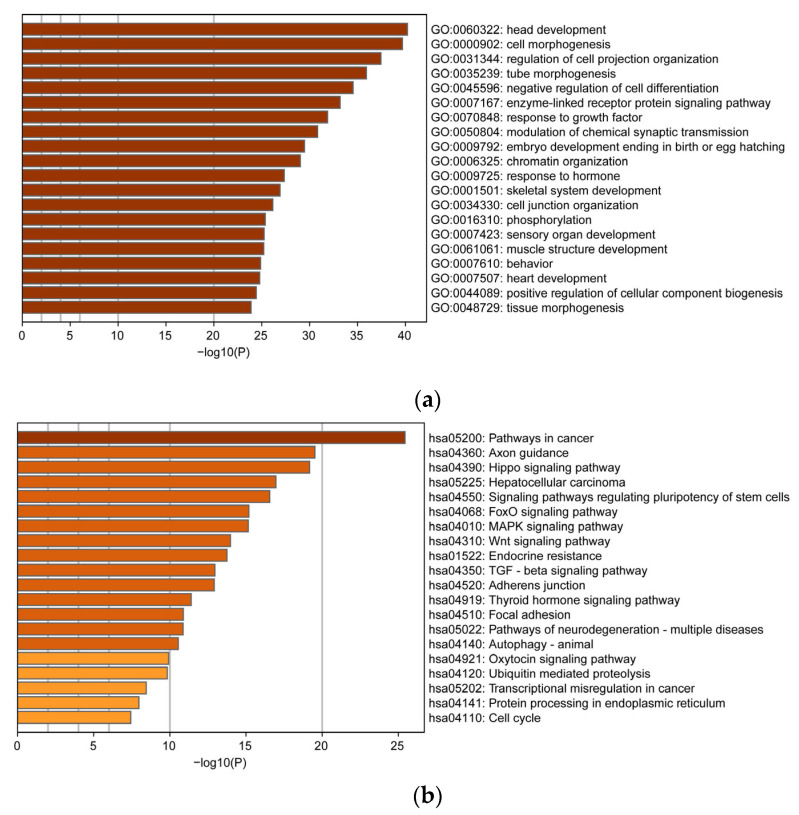
Functional enrichment analysis of DE (differentially expressed) miRNA target genes: (**a**) gene ontology enrichment analysis; (**b**) Kyoto Encyclopedia of Genes and Genomes pathway enrichment analysis. Note: In the figure, different colors represent the enrichment levels of DE miRNAs. The higher the enrichment level, the darker the color.

As shown in Figure 3a and Table S5, the target genes of the identified DE miRNAs were found to be involved in various biological processes. These include the negative regulation of cell differentiation (GO:0045596), the regulation of enzyme-linked receptor protein signaling pathways (GO:0007167), the response to growth factors (GO:0070848), and also the regulation of responses to hormones (GO:0009725). Furthermore, the KEGG pathway enrichment analysis revealed that the miRNAs were associated with pathways related to multiple diseases, namely the pathways in cancer, endocrine resistance, and neurodegeneration. Additionally, they were involved in signaling pathways related to MAPK, Wnt, Hippo, and FoxO (Figure 3b, Table S5). These findings indicate that these miRNAs may have critical functions in giant panda development, including disease regulation and crucial signaling pathways.

## 4. Discussion

As a national-level protected animal in China, researchers have been working on the immune and disease aspects of giant pandas to better protect them. Blood is a significant component of the animal’s immune system and is important in the immune response [24]. Dynamic changes in blood composition reflect the state of cells, tissues, organs, and the body as a whole, and these changes also reflect existing diseases in the organism or the onset of future diseases [25]. MicroRNAs are present in a variety of bodily fluids and are stable. Therefore, miRNAs in the blood can also be used as a biomarker to reflect pathophysiological conditions in the tissue of origin [26]. In our study, we performed small-RNA sequencing on the blood of nine giant pandas at three age stages, subadult (1.5Y–2Y), subadult (2.5Y–3Y), and adult (8Y–15Y), and clarified the expression profiles of miRNAs in their blood.

Chen et al. discovered that RBCs were the major contributors to miRNA expression in whole blood [27]. MiR-486, miR-144, and miR-451, which are abundant in red blood cells (RBCs), are involved in erythropoiesis and disease occurrence [28]. Our results show that miR-486-5p exhibited the highest abundance in giant panda blood at all ages. Consistent with our study, Wang et al. found that miR-486-5p ranked first among all miRNA abundances in the blood of giant pandas during pregnancy and lactation. The data of Yang M.Y. et al. show that the abundance of miR-486-5p in giant panda blood was in the top five in both the subadult (1.5Y–2Y) and adult ages. miR-486 could mediate hematopoietic cell growth and survival by regulating AKT signaling and FOXO1 expression [29]. These results suggest that miR-486-5p may be critical in the physiological function of giant panda blood cells. Our results also show that the top ten most highly expressed miRNAs in the three age stages were similar overall. Among them, the top three most highly expressed miRNAs were the same at all three age stages, which were miR-486-5p, miR-92a-3p, and miR-191-5p. Huili Lia et al. showed that miR-92a-3p might increase the γ-globin level and reduce oxidative stress and apoptosis in erythroid precursor cells by downregulating *BCL11A* [30]. miR-191-5p, on the other hand, was found to be involved in an inflammation-associated lncRNA-miRNA-mRNA regulatory axis, which is closely related to interleukin family signaling, neutrophil degranulation, adaptive immunity, and cell adhesion pathways [31].

We identified a total of 683 mature miRNAs in the blood of giant pandas of three ages and screened for DE miRNAs. Some DE miRNAs have previously been shown to be associated with immune responses in animal organisms. For example, miR-150-3p can regulate the innate immune response by inhibiting the *Trim14*/NF-κB/*IFNβ* axis. Thus, miR-150-3p can be a potential biomarker for inflammation diagnosis, treatment, and prognosis [32]. Xue Taobai et al. found that miR-28-3p in peripheral blood single-nucleated cells targeted a sequence in viral gag/pol genomic viral mRNA and was able to reduce viral replication and gene expression of human T-cell leukemia virus type 1 (*HTLV-1*) [33]. miR-7-5p in plasma exosomes can inhibit T-lymphocyte apoptosis in patients with sepsis by directly targeting and regulating the Bad gene in the cGMP-PKG signaling pathway, as well as by upregulating the mRNA and protein levels of the anti-apoptotic gene, *Bcl-2*, to attenuate immunosuppression in patients with sepsis [34].

Our results show that the target genes of DE miRNAs were mainly enriched in the “negative regulation of cell differentiation”, “enzyme-linked receptor protein signaling pathway”, and “phosphorylation” GO category. In addition, the KEGG pathway analysis showed that these target genes were mainly enriched in MAPK, Hippo, and FoXO signaling pathways. Previous studies have shown that MAPK is closely related to cytokine and protease levels, cell cycle, motility, and metabolism and can be a major effective target for treating various cancers and inflammatory diseases [35]. It has also been found that overexpression of miR-101-3p in peripheral blood mononuclear cells (PBMCs) can attenuate inflammatory responses by targeting and regulating MAPK1 expression and blocking the NF-κB pathway [36]. Deregulation of the Hippo signaling pathway leads to apoptosis-resistant and migrating tumors, whereas miR-9-3p, which resulted from the screening of our study, was found to target and regulate the expression of pro-oncogenic factor TAZ downstream of the Hippo pathway [37]. FOXO plays a key role in regulating cell cycle progression and cell survival, and the Akt-FoXO signaling axis is an essential signal for controlling the cellular initiation, proliferation, and differentiation of endothelial progenitor cells (EPCs). Xiaolong Du found that miR-150 in peripheral blood was able to regulate cellular progenitor cells (EPCs) by directly targeting the Akt-FoXO signaling axis and regulating the proliferation and differentiation of EPCs, thereby promoting the lysis of deep vein thrombosis [38,39].

This study delved into miRNA expression within the whole blood of giant pandas at different developmental stages. Notably, the identified miRNAs mirrored the comprehensive miRNA profile originating from diverse sources, such as different blood cells, exosomes, and plasma. Future studies should aim to analyze sorted cell populations to provide more precise insights into the cell-specific expression patterns of miRNAs.

## 5. Conclusions

In conclusion, we identified a total of 393 known miRNAs, 219 conserved miRNAs, and 71 novel miRNAs in the blood of giant pandas of different ages using small-RNA sequencing. Ail-miR-486-5p had the highest abundance in the blood of giant pandas of all ages. The screened DE miRNAs were mainly involved in the negative regulation of cell differentiation and the regulation of enzyme-linked receptor protein signaling pathways. They were enriched in MAPK, Hippo, and Wnt signaling pathways. Our findings not only expand the range of blood miRNAs of giant pandas across different developmental stages but also contribute to a deeper understanding of the roles of miRNAs in blood immunity and the regulation of blood cell physiological functions in giant pandas. The data obtained from this study provide an essential reference for the health assessment of giant pandas from the perspective of miRNAs.

## Figures and Tables

**Table 1:** Mapping the clean reads to the reference genome.

Sample	Raw Reads	Clean Reads	Mapped	Mapped Ratio (%)
Subadult(2.5Y–3Y)_A	13,761,185	13,744,582	12,511,010	91.0
Subadult(2.5Y–3Y)_B	12,579,034	12,550,467	11,877,863	94.6
Subadult(2.5Y–3Y)_C	15,105,867	15,094,790	14,260,774	94.5
Subadult(1.5Y–2Y)_A	13,693,555	13,669,546	12,837,830	93.9
Subadult(1.5Y–2Y)_B	11,080,391	11,054,356	10,408,657	94.2
Subadult(1.5Y–2Y)_C	12,533,000	12,426,921	11,234,412	90.4
Adult_A	11,683,594	11,671,006	11,045,728	94.6
Adult_B	13,910,688	13,827,144	13,187,016	95.4
Adult_C	13,940,303	13,922,500	13,261,560	95.3

**Table 2:** Giant panda miRNAs identified in nine sRNA libraries.

Type	Pre-miRNAs	miRNA-5p	miRNA-3p	Both	Mature miRNAs
Known miRNAs	314	63	56	137	393
Conserved miRNAs	643	94	99	13	219
Novel miRNAs	87	21	30	10	71

## Data Availability

The raw sequence data reported in this paper have been deposited in the Genome Sequence Archive (Genomics, Proteomics & Bioinformatics 2021) in the National Genomics Data Center (Nucleic Acids Res 2024), the China National Center for Bioinformation/Beijing Institute of Genomics, Chinese Academy of Sciences (GSA: CRA013317), and are publicly accessible at https://ngdc.cncb.ac.cn/gsa/browse/CRA013317 (accessed on 17 February 2025).

## Supplementary Materials

The following supporting information can be downloaded at https://www.mdpi.com/article/10.3390/genes16030243/s1, Figure S1: Length distributions of small RNAs in nine libraries; Table S1: Known miRNAs; Table S2: Conserved miRNAs; Table S3: Novel miRNAs; Table S4: List of differentially expressed miRNAs; Table S5: Function analysis for DE miRNAs.
