# Supplementary figures and images for: MicroRNA Expression Profile Analysis in Blood During Giant Panda (Ailuropoda melanoleuca) Growth and Development
Source: Genes (Basel). 2025 Feb 20;16(3):243. doi: 10.3390/genes16030243 (PMC11942061; doi:10.3390/genes16030243)

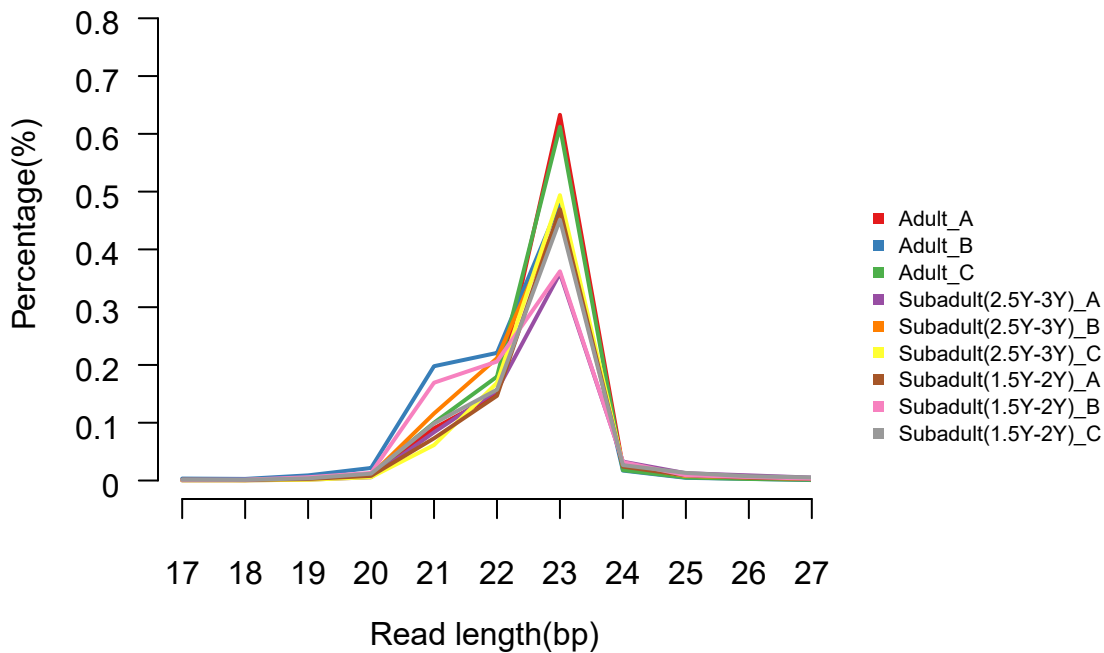

Supplement: Supplementary file 1 [file genes-16-00243-s001.zip › figure_S1_Length_distributions_of_small_RNAs_in_nine_libraries.pdf]
